# Editorial: Advances in pediatric rehabilitation clinical trials: design, methods, and analysis

**DOI:** 10.3389/fped.2025.1748529

**Published:** 2025-12-03

**Authors:** Stephanie C. DeLuca, Amy R. Darragh, Jill Heathcock

**Affiliations:** 1 Virginia Tech, Fralin Biomedical Research Institute at VTC, Roanoke, VA, United States; 2 College of Health Professions, Virginia Commonwealth University, Richmond, VA, United States; 3 School of Health and Rehabilitation Sciences, The Ohio State University, Columbus, OH, United States

**Keywords:** pediatric rehabilitation, disability, clinical trials, rehabilitation research, pediatric rehabilitatin research, development, childhood-onset disability

The work in this Research Topic represents critical topics in pediatric rehabilitation: measurement, participation, precision rehabilitation/habilitation, rehabilitation complexity, and lifespan considerations. Pediatric rehabilitation researchers confront challenges as they design and implement studies because of the many variables that influence rehabilitation intervention outcomes, such as child growth and development, family ecosystems, community systems, disease processes and even world events. As editors we, Stephanie C. DeLuca (Virginia Tech), Jill Heathcock (The Ohio State University), and Amy Darragh (Virginia Commonwealth University), would like to express our deep gratitude to the 86 contributing authors who chose to publish their work in our Research Topics. The presented work covers areas broadly summarized around measurement, intervention, and theory. The articles describe multiple topics and challenges in pediatric rehabilitation research and will provide guidance for the future rehabilitation research, ultimately impacting the lives of many children with disabilities and their families.

One area considered in this Research Topic was measurement. Pediatric rehabilitation measures must be reliable and valid, and they must measure what matters to children and families. Our authors present an array of measures that reflect measurement success and challenges in rehabilitation research. Their works represents efforts to move the field forward by developing tools that detect change in real world settings, impact participation in daily activities, assess subtle preferences and abilities, and advance precision rehabilitation. Petruccelli et al., used advanced technology to measure physical recovery in a real-world environment among children recovering from critical illness. Sarsak and Rushton focused on measuring wheelchair confidence in children using manual wheelchairs because low confidence in wheelchair use is associated with less participation in daily activity. Mulrenin et al., emphasize the importance of detecting subtle differences in preferences and abilities in children with complex medical conditions. Skorup et al., articulate the importance of precision measurement in intervention: specifically, how the measurement and contributions of understanding active ingredients can inform variations in interventions (e.g., motor error, the study's focus) based on individual child characteristics. Collectively, these articles present the intersection of psychometrically sound measures that facilitate measurement of meaningful, relevant, and important constructs to children and families.

This Research Topic included three intervention studies, Vacchini et al., Brennan et al., and Kemp et al., that identify the importance of individualized interventions that focus on real world impact. Vacchini et al. present a protocol in which they focus on family- and child-identified goal and activity preferences, environmental context, and an intervention that is tailored for the abilities of each child. Brennan et al. conducted a study of adaptive cycling for children with disabilities. Similarly, they focused on child-centered goals and delivered the intervention in a contextually-relevant environment (school). The study focused on improving the child's health and participation, using a functional and age-appropriate activity that improved physical activity, and, in this case, happiness. Relatedly, Kemp et al. delivered a personalized intervention with individualized goals in a community environment (pool in a local school) that emphasized participation in an age-appropriate and functional activity, swimming. The study focused on improving water competency with the long-term goal of drowning prevention, a leading cause of death for children with autism. Thematically, these studies demonstrate the importance of research that is family- and child-centered, individualized and relevant, and focused on activity and participation.

Finally, three articles presented frameworks for considering or reconsidering approaches to pediatric rehabilitation to ensure all children with disabilities have access to and receive the care they deserve. Bican et al. identified the serious and wide ranging barriers to care experienced by families of children with disabilities in rural communities, such as access to and waitlists for specialists, distance to providers, costs of gas, care for siblings, and transportation, and recommend policy change and continued research to ensure children living in under-resourced and/or rural areas in the U.S. receive rehabilitation services that support function and participation. Cai et al. assessed decision making for early pulmonary rehabilitation (PR) in the PICU and identified multiple intrinsic and extrinsic factors that impact delivery of early PR including lack of knowledge and training in PR, lack of standards of care for PR, and then experiences of caring for critically ill children. Finally, Ramey et al. present an interdisciplinary developmental framework they titled the Interdisciplinary Monitoring, Planning, and Caring for the Total-Child – Together (IMPACT2) to guide inter-community interaction towards effective pediatric rehabilitation. All identify the gaps in policy, access, education, training, and context that impact service delivery for children with disabilities, the result of which can have lifelong consequences.

The need for specialized and collaborative work across disciplines is greater than ever as rehabilitation professionals seek to be positive change agents in the lives of children and families. In pediatric rehabilitation, interventions occur at single points or short periods in time, but there is an ever-increasing knowledge that these points occur in a mass of interacting trajectories of influence. Each child's pediatric rehabilitation journey is an interacting web of these variables that travel with them across time as they often complete a multitude of interventions. The choice of these interventions and their success are manifestly defined by that child and the family. Almost by definition, this means that rehabilitation must consider “meaningful” change as a dynamic and “individualized” process. Measures of rehabilitation intervention efficacy, harm, and effectiveness all occur within complex contextual relationships that cannot be set aside. Thus, the intricacies of understanding precision rehabilitation or even a single intervention exceed those typically encountered in health research addressing chronic conditions. Pediatric rehabilitation researchers and clinicians are often in positions that require them to challenge the status quo because of these complexities. These issues are just what we as editor sought to address, and the authors answered our call. We and the authors are challenging the long and pervasive history of clinical practice and scientific inquiry that emphasized of biomarkers, singular primary outcome measures, and expert opinions while often excluding the opinions and perspectives of families and individuals with disabilities – the true experts. We are grateful to all who rose to aid us in giving voice to these issues.

